# Steroid treatment as anti-inflammatory and neuroprotective agent following out-of-hospital cardiac arrest: a randomized clinical trial

**DOI:** 10.1186/s13063-022-06838-0

**Published:** 2022-11-22

**Authors:** Laust Emil Roelsgaard Obling, Rasmus Paulin Beske, Sebastian Wiberg, Fredrik Folke, Jacob Eifer Moeller, Jesper Kjaergaard, Christian Hassager

**Affiliations:** 1 Department of Cardiology, The Heart Centre, Copenhagen, Denmark; 2 University Hospital - Rigshospitalet, Copenhagen, Denmark; 3 Department of Cardiothoracic Anesthesiology, The Heart Centre, Copenhagen University Hospital – Rigshospitalet, Copenhagen, Denmark; 4 Department of Cardiology, Copenhagen University Hospital - Herlev-Gentofte Hospital, Copenhagen, Denmark; 5 Copenhagen Emergency Medical Services, University of Copenhagen, Copenhagen, Denmark

**Keywords:** Acute cardiac care, Out-of-hospital cardiac arrest, Pre-hospital intervention, Randomized controlled trial, Post cardiac arrest syndrome, Inflammation, Neuroprotection, Steroid, Hemodynamics

## Abstract

**Background:**

Patients resuscitated from out-of-hospital cardiac arrest (OHCA) have a high morbidity and mortality risk and often develop post-cardiac arrest syndrome (PCAS) involving systemic inflammation. The severity of the inflammatory response is associated with adverse outcome, with anoxic irreversible brain injury as the leading cause of death following resuscitated OHCA. The study aimed to investigate the anti-inflammatory and neuroprotective effect of pre-hospital administration of a high-dose glucocorticoid following OHCA.

**Methods:**

The study is an investigator-initiated, randomized, multicenter, single-blinded, placebo-controlled, clinical trial. Inclusion will continue until one hundred twenty unconscious OHCA patients surviving a minimum of 72 h are randomized. Intervention is a 1:1 randomization to an infusion of methylprednisolone 250 mg following a minimum of 5 min of sustained return of spontaneous circulation in the pre-hospital setting. Methylprednisolone will be given as a bolus infusion of 1 × 250 mg (1 × 4 mL) over a period of 5 min. Patients allocated to placebo will receive 4 mL of isotonic saline (NaCl 0.9%). Main eligibility criteria are OHCA of presumed cardiac cause, age ≥ 18 years, Glasgow Coma Scale ≤ 8, and sustained ROSC for at least 5 min. Co-primary endpoint: Reduction of interleukin-6 and neuron-specific-enolase. Secondary endpoints: Markers of inflammation, brain, cardiac, kidney and liver damage, hemodynamic and hemostatic function, safety, neurological function at follow-up, and mortality. A research biobank is set up with blood samples taken daily during the first 72 h from hospitalization to evaluate primary and secondary endpoints.

**Discussion:**

We hypothesize that early anti-inflammatory steroid treatment in the pre-hospital setting can mitigate the progression of PCAS following resuscitated OHCA. Primary endpoints will be assessed through analyses of biomarkers for inflammation and neurological damage taken during the first 72 h of admission.

**Trial registration:**

EudraCT number: 2020-000855-11; submitted March 30, 2020

ClinicalTrials.gov Identifier: NCT04624776; submitted October 12, 2020, first posted November 10, 2020

## Administrative information

Note: this protocol’s numbers in curly brackets refer to SPIRIT checklist item numbers. The order of the items has been modified to group similar items (see http://www.equator-network.org/reporting-guidelines/spirit-2013-statement-defining-standard-protocol-items-for-clinical-trials/).

**Table:** 

Title {1}	Steroid treatment as anti-inflammatory and neuroprotective agent following out-of-hospital cardiac arrest. A randomized clinical trial.
Trial registration {2a and 2b}.	ClinicalTrials.gov Identifier: NCT04624776 EudraCT: 2020–000,855-11
Protocol version {3}	Version: 3.2 of November 11^th^, 2021
Funding {4}	The study is supported by a grant from Novo Nordisk Foundation (NNF20OC0064043) LOs salary is supported by the Rigshospitalet Research Foundation RBs salary is supported by the grant from Novo Nordisk Foundation (NNF20OC0064043) CHs salary is supported by an unrestricted grant from Lundbeck Foundation (R186-2015–2132) JK is supported by an unrestricted grant from Novo Nordisk Foundation (NNF17OC0028706) for research in post-cardiac arrest management FF is supported by a research grant from Novo Nordisk Foundation (NNF19OC0055142)
Author details {5a}	LO, RB, JEM, JK and CH: Department of Cardiology, The Heart Centre, Copenhagen University Hospital—Rigshospitalet, Copenhagen, Denmark SW: Department of Cardiothoracic Anesthesiology, The Heart Centre, Copenhagen University Hospital – Rigshospitalet, Copenhagen, Denmark FF: Department of Cardiology, Copenhagen University Hospital—Herlev-Gentofte Hospital, Copenhagen, Denmark Copenhagen Emergency Medical Services, University of Copenhagen – Copenhagen, Denmark
Name and contact information for the trial sponsor {5b}	Trial sponsor-investigator: Christian Hassager, Professor, MD, DMSc Dept. of Cardiology, The Heart Centre, Copenhagen University Hospital—Rigshospitalet, Copenhagen, Denmark Email: christian.hassager@regionh.dk
Role of sponsor {5c}	This is a sponsor-investigator initiated study, and the sponsor-investigator maintains authority over all aspects of the trial including, design, management, interpretation of results and publication.

## Introduction

### Background and rationale {6a}

Each year, approximately 5000 individuals suffer from out-of-hospital cardiac arrest (OHCA) in Denmark, and despite an improved prognosis, 30-day mortality is approximately 85% [1]. For unconscious OHCA patients admitted to an intensive care unit (ICU) after successful resuscitation, the 30-day mortality remains higher than 50% [2, 3].

Unconscious patients resuscitated from OHCA often develop a complicated systemic response, called post-cardiac arrest syndrome (PCAS) [4, 5]. PCAS consists of four interacting components: (1) ischemic/reperfusion brain injury with possible development of brain edema, (2) myocardial dysfunction, (3) a systemic inflammatory response, and (4) persistent stress from the triggering cause of the cardiac arrest, e.g., acute myocardial infarction (AMI) [4]. PCAS progresses during the first days in unconscious patients following resuscitated cardiac arrest, and the treatment aims to reduce neurologic injury by targeted temperature management, circulatory support with vasopressors, and inotropics or mechanical devices as well as identification and treatment of reversible causes to the cardiac arrest, e.g., acute revascularization of an AMI [5, 6].

Several studies have shown that the systemic inflammatory response is associated with a high risk of poor outcome following OHCA in unconscious patients [7–9]. Despite this, no specific treatment targets this complicated and life-threatening inflammatory response, and guidelines remain inconclusive in this field.

Anoxic irreversible brain injury remains the leading cause of death in unconscious patients following resuscitated OHCA [10, 11], and it is thought to develop due to neuron apoptosis and reperfusion/ischemic injury [4, 12]. Inhibiting the causes of the anoxic brain injury in the early stages following resuscitation from OHCA may therefore be key to optimizing post-cardiac arrest care.

Inflammatory markers associated with poor outcome include interleukin (IL) 6, high-sensitivity C-reactive protein (hsCRP), leucocytes, IL-1b, IL-10, IL-13, tumor-necrosis factor (TNF) α, and procalcitonin [7, 13, 14].

The biomarker neuron-specific enolase (NSE) correlates to neuron damage in the blood stream and has a strong predictive value for poor outcome following OHCA [15, 16].

Following resuscitated OHCA, the function of the adrenal gland is compromised due to global ischemia, and accordingly, reduced levels of the steroid glucocorticoid are produced [17, 18]. Glucocorticoid has an important role in several physiologic processes including a systemic anti-inflammatory response [19, 20]. As a result, resuscitated cardiac arrest patients are affected by a severe inflammatory response, while the body’s natural defense mechanism to modulate inflammation is suppressed.

Systemic treatment with glucocorticoids serves as an anti-inflammatory mediator. It counteracts acute microcirculation injury and free radical formation, resulting in diminished vasodilation and reduction of edema, e.g., brain edema [20–22]. These anti-inflammatory effects are mediated through a slow genomic response (hours) and a rapid non-genomic response (seconds/minutes) [23]. Two former small studies have shown signs of improved survival and neurologic outcome among patients who were given injections with glucocorticoids after in-hospital cardiac arrest (IHCA) [24, 25] and a similar contemporary study found an association between glucocorticoids and higher rates of return of spontaneous circulation (ROSC) but with no impact on survival [26]. The incidence of adverse events was not higher in patients receiving glucocorticoids in the three studies. Long-term treatment with glucocorticoids is associated with a series of side effects, whereas short-term treatment only has limited side effects [27]. Short-term systemic treatment with glucocorticoids could therefore be an essential and safe factor in the acute treatment of resuscitated cardiac arrest patients that could potentially improve survival and neurological outcome [22].

In summary, PCAS is a life-threatening condition following resuscitated OHCA that involves a systemic inflammatory component. PCAS and the level of inflammation are associated with increased mortality and poor neurological outcome. Inhibition of this inflammatory response may have an important, yet relatively unknown, role in post-cardiac arrest care. Early treatment following resuscitation with the anti-inflammatory glucocorticoid methylprednisolone in the pre-hospital setting could mitigate inflammation through non-genomic pathways and thereby prevent further development of PCAS, leading to improved outcome.

### Objectives {7}

#### Primary objective

The primary objective is to determine the efficacy of methylprednisolone compared with placebo administered after ROSC on outcome parameters: daily IL-6 and NSE levels from admission to 72 h later, in comatose, resuscitated OHCA patients.

#### Secondary objectives

The secondary objectives are to determine the effects of methylprednisolone on markers of inflammation, cardiac protection, neuroprotection, renal protection and endothelial protection, clinical endpoints including survival and neurological outcome, and safety. Quantification of secondary objects is specified in the “Outcomes {12}” section.

#### Hypothesis

Bolus infusion of 250 mg methylprednisolone in the pre-hospital setting will inhibit the systemic inflammatory response and reduce biomarkers of the degree of cerebral injury in comatose, resuscitated OHCA patients.

### Trial design {8}

The study was designed as an investigator-initiated, 1:1 randomized, multicenter, blinded, placebo-controlled phase II clinical superiority trial.

## Methods: participants, interventions, and outcomes

### Study setting {9}

Multicenter study carried out at the following sites:

1. The Cardiac ICU at the Department of Cardiology, The Heart Centre, Copenhagen University Hospital—Rigshospitalet, Copenhagen
2. The ICU, Copenhagen University Hospital—Gentofte Hospital, Copenhagen
3. The Emergency Medical Services of the Capital Region of Denmark—Copenhagen

### Eligibility criteria {10}

All resuscitated OHCA patients in the Capital Region of Denmark are screened for eligibility. The screening is performed by the on-duty physician manning the mobile critical care unit (CCU). The Danish Cardiac Arrest Registry will be used as a screening log during the trial. Following successful screening, the patient will be included, and study drug will be given. Inclusion in the STEROHCA trial does not prohibit participation in other trials.

#### Inclusion criteria

1. Age ≥ 18 years
2. OHCA of presumed cardiac cause
3. Unconsciousness (GCS ≤ 8) upon pre-hospital randomization
4. Sustained ROSC for at least 5 min
5. Randomization and start of study medicine infusion within 30 min of sustained ROSC

#### Exclusion criteria

1. Advanced life support termination-of-resuscitation (TOR) exclusion criteria [28, 29].
2. Asystole as primary electrocardiogram (ECG) rhythm
3. Women of childbearing potential
4. Known therapy limitation (known decision made of no resuscitation or intensive therapy)
5. Known allergy to methylprednisolone
6. Known pre-arrest modified Rankin Scale (mRS) score of 4–5
7. Temperature upon randomization < 30° C
8.  > 30 min to sustained ROSC

### Who will take informed consent? {26a}

The trial will be conducted in accordance with the Declaration of Helsinki and follow European and national legislation on medical research in emergency situations with subjects temporarily incapacitated. Since subjects are incapacitated, they will not be able to provide informed consent prior to enrollment. According to Danish legislation, a proxy consent from a legal surrogate (trial guardian) is required on behalf of the patient prior to inclusion. The trial guardian providing informed consent will be a medical doctor with no involvement in the patient treatment in this trial. The consent will be filled out through the encrypted database program REDCap. Further, informed consent from the next of kin will be obtained at the earliest time possible. The latter consent will be obtained by the physician on call or a dedicated team of research personnel, all medical doctors. A consent from a secondary trial guardian will be obtained following admission of the patient in REDCap. Approvals from the relevant authorities can be seen in section {24}.

### Additional consent provisions for collection and use of participant data and biological specimens {26b}

Approval from the local ethical committee will be needed for ancillary studies of patient data or biological specimens unless this is waived based on prior approvals or the design of the studies.

### Interventions {11}

#### Explanation for the choice of comparators {6b}

We have chosen a single bolus infusion of 250 mg methylprednisolone (Solumedrol) for attenuation of the inflammatory response seen following OHCA. Pulse doses of methylprednisolone (≥ 250 mg) are widely used in the treatment of conditions such as prevention of organ rejection after transplantation, rheumatic diseases with acute deterioration, multiple sclerosis, and acute injury of the bone marrow [30]. At methylprednisolone doses above 250 mg, infusion time should be above 30 min, whereas doses below 250 mg can be completed within 5 min, according to the Danish summary of product characteristics. Placebo is chosen as comparator since there is no known effective treatment targeting inflammation and neuroprotection in resuscitated OHCA patients.

#### Intervention description {11a}

Patients allocated to methylprednisolone will receive a bolus infusion of 250 mg over 5 min. The study drug is prepared by a simple shaking-mechanism where 2 × 125 mg/2 mL of methylprednisolone powder is mixed preservative-free to a total volume of 4 mL. Patients allocated to placebo will receive a 4 mL infusion of isotonic saline. Infusion of study drug or placebo will be made as soon as possible following 5 min of stable ROSC in the pre-hospital setting.

#### Criteria for discontinuing or modifying allocated interventions {11b}

If the next of kin or the trial guardian of a patient refuses or withdraws consent to participate in the study, the patient will be withdrawn from the trial without prejudice to future medical care. Further, if the patient refuses or withdraws consent, when able to, no study observations will be made from the date of request and the patient will be removed from the study.

A patient can be withdrawn from the study by the sponsor-investigator if any of the following occurs: (1) significant intercurrent illness, (2) patient refusal to continue observations, and (3) investigator decides that termination is in the best medical interest of the patient.

If withdrawal of consent occurs, observations will be completed to the date of withdrawal and a final evaluation of the patient’s withdrawal will be made at this point with an explanation for the withdrawal. If withdrawal is due to an adverse event, the patient will be followed until the adverse event is stabilized or resolved.

#### Strategies to improve adherence to interventions {11c}

All trial guardians and physicians including patients have relevant knowledge of the cardiological specialty and knowledge of the study from an obligatory instruction video and the handing out of the study protocol, protocol summaries, and pocket cards. None of these physicians are involved with the primary care of the patient following admission. The study medicine is a single bolus infusion administered over 5 min, and there is a low risk of missing or delaying the intervention following screening of the patient.

In-hospital personnel involved in the primary care of the patient, i.e., doctors and nurses, have relevant knowledge of the study through the instruction video and written instructions and have been trained in data collection study specific procedures.

#### Relevant concomitant care permitted or prohibited during the trial {11d}

According to temporary guidelines, all patients included in the study will be treated with standard therapies for OHCA, including targeted temperature management at 36 degrees, vasopressors and/or inotropes if needed and prophylactic antibiotics. Necessary cardiac interventions will not be delayed by the trial intervention. The specific care of all patients is left to the discretion of the treating physician.

#### Provisions for post-trial care {30}

Included patients will be insured by the Patient Compensation Association (the health system responsible for the trial sites, “Rigshospitalet” and “Gentofte Hospital”).

### Outcomes {12}

#### Primary outcome

The primary outcome is the repeated daily measurements of IL-6 and NSE at admission and the following 24, 48, and 72 h after admission.

#### Secondary outcomes

Markers of inflammation: hsCRP, leucocyte- and differential count, plasma cytokine levels (IL1b, IL-2, IL-4, IL-5, IL-7, IL-8, IL-10, IL-12, IL-13, IL-17A, GM-CSF, G-CSF, MCP-1, MIP-1beta, IFN-g and TNF-α), and procalcitonin measured daily the first 3 days from admission.

Markers of kidney and hepatic injury: Daily creatinine levels the first 3 days from admission. Daily measurements of ALAT, ASAT, ALP, bilirubin, and INR the first 3 days from admission.

Markers of the coagulation system (only at the site “Rigshospitalet”): Plasma fibrinogen the first three days from admission, and thromboelastography (TEG) at admission and at 48 h.

Protein and enzymatic protection: Daily proteomics and metabolomics samples the first 3 days from admission.

Hemodynamics: Daily evaluation by Swan-Ganz catheter (only at “Rigshospitalet”), bihourly analyses of arterial blood gasses the first 36 h and transthoracic echocardiogram (TTE) measured at time of admission and before hospital discharge.

Neuroprotection: Daily measurements of Tau and NfL levels the first 3 days from admission.

Cardiac protection: Measurements of TnT (at “Rigshospitalet”), TnI (at “Gentofte Hospital”), and CKMB levels at admission and the following 6, 12, 24, 36, 48, and 72 h of admission.

Clinical endpoints: Daily Sequential Organ Failure Assessment (SOFA) scores the first 3 days from admission. Survival and neurological outcome, decided by CPC and mRS score after 5 days, at discharge from the intensive ward and the hospital and following a minimum of 180 days from discharge through telephone interview.

Follow-up at 90 days following discharge in an ambulatory setting: MoCA-score for assessment of cognitive function and patients will be screened for quality of life, anxiety, depression, and the return to daily living with their nearest relatives.

Safety: Cumulated daily incidence of any adverse event (AE) the first 7 days, including “severe adverse event” (SAE), and “adverse event with believed relation to the study medicine” (SUSAR).

### Participant timeline {13}

**Fig. 1:**
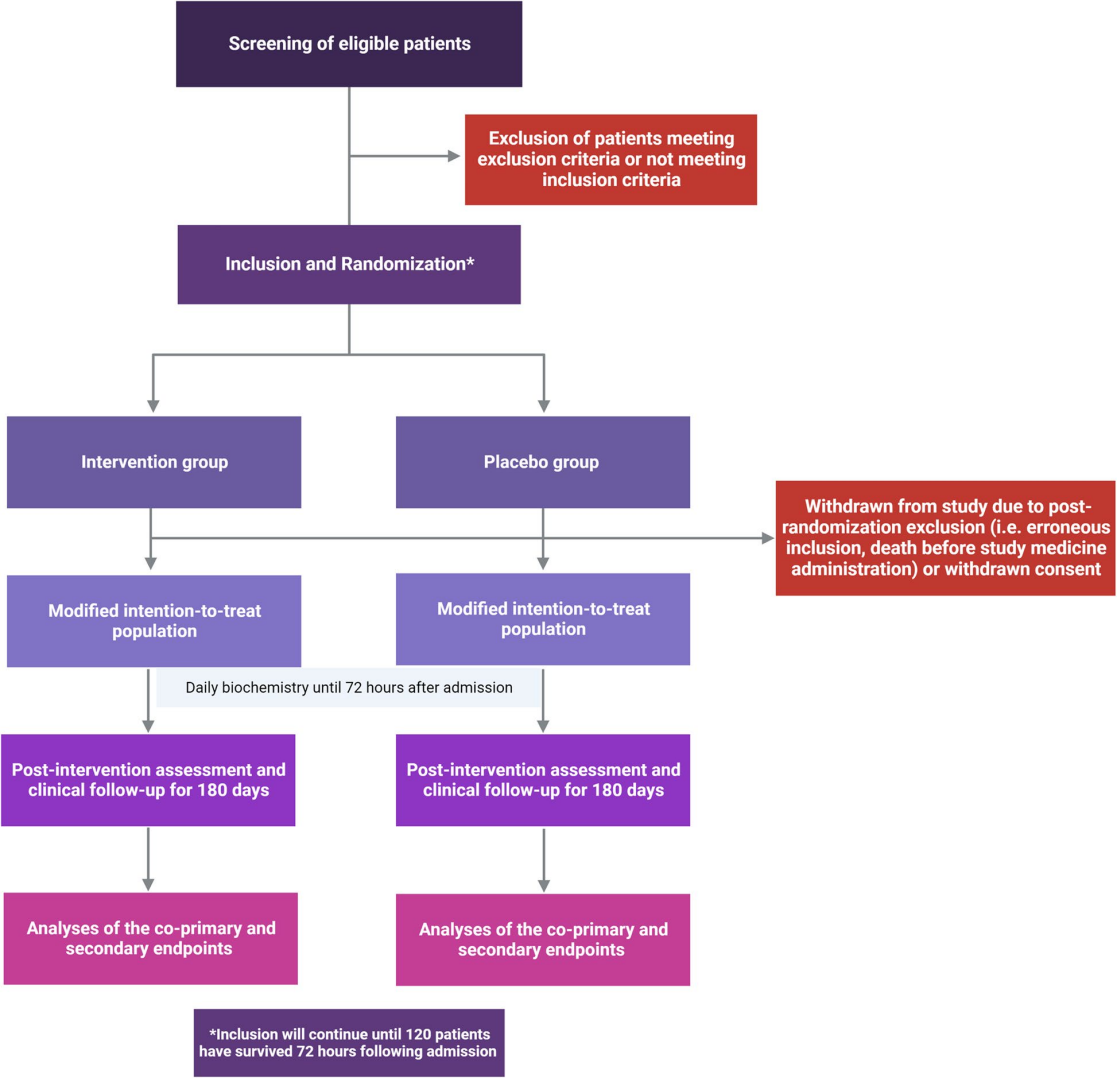
Flow chart for the trial

Besides the section presenting outcomes above ({12}), a flow chart for the trial is presented in Fig. 1, and a time schedule for the trial is presented as a schematic diagram in Table 1.

**Table 1:** Time schedule for the trial

	**Trial period**							
	**Enrolment**	**Post-allocation**						**Follow-up**
**Timepoint**	*0 h*	*6 h*	*12 h*	*24 h*	*36 h*	*48 h*	*72 h*	*Days 90 and 180*
**Enrolment**								
**Eligibility screen**	x							
**Informed consent**	Trial guardian and next of kin							Patient as soon as possible
**Randomization**	x							
**Interventions**								
**Infusion of study drug**	x							
**Assessments**								
**Biochemistry**^**a**^	x	x	x	x	x	x	x	
**Biobank samples**	x			x		x	x	
**ECG**	x	x	x	x	x	x	x	
**Swan-Ganz-based measurements**	x	x	x	x	x	x	x	
**Echocardiography**				Day 1				Before discharge
**ABG and VBG**^**b**^	x	x	x	x	x	x	x	
**SOFA score**				x		x	x	
**CPC and mRS score**								x
**MoCA score**								Only day 90

*ABG* Arterial blood gas, *CPC* Cerebral Performance Category, *ECG* Electrocardiogram, *mRS* Modified Rankin Score, *MoCA* Montreal Cognitive Assessment, *SOFA* Sequential Organ Failure Assessment, *VBG* Venous blood gas

^a^Details in outcomes section in the manuscript

^b^Additional blood gasses are taken bihourly until 12 h and at 18 h

### Sample size {14}

The trial is powered at the co-primary endpoint. We chose a priori to power the trial at the “weakest” of the two endpoints, ensuring that we would have sufficient power for both endpoints. As we were not able to find data regarding the effect of methylprednisolone on IL-6- or NSE levels from admission, we chose to power the trial towards a single measurement drawn 48 h after admission. With the assumption that methylprednisolone would reduce IL-6 levels by 20%, the trial would achieve a power of 0.90 at an alpha-level of 0.025 if 112 patients were included. With the assumption that methylprednisolone would reduce NSE levels by 20%, the trial would achieve a power of 0.90 at an alpha-level of 0.025 if 114 patients were included.

Based on the above, inclusion of 120 patients would be sufficient to adjust for missingness due to withdrawn consent, though we estimated from previous studies that approximately 20% of resuscitated OHCA patients would die within 72 h, i.e., before complete assessment of the co-primary endpoint. Further, due to the acute nature of the trial, we expect 10% of post-randomization exclusions and/or patients where consent is not obtained. To achieve a complete set of blood samples from 120 patients, we, therefore, plan to include 1.30 × 120 = 156 patients. We will however stop the trial before if our target at 120 patients is reached.

### Recruitment {15}

The study includes an enrolment period of up to 18 months, followed by a follow-up period of 6 months and analyses of paraclinical results another 12 months. The expected study period is based on previous clinical studies in OHCA patients at our department, where approximately 150 patients are included each year (including OHCA patients admitted from outside the Capital Region of Denmark).

## Methods: assignment of interventions

### Allocation {16}

#### Sequence generation {16a}

The pharmacy of the Capital of Region of Denmark will prepare, box, and label the study medicine and the placebo ampoules. The pharmacy is approved by the Danish Health authorities. Randomization will be generated by using the website randomization.com (http://www.randomization.com) with allocation in a 1:1 fashion with active study medicine and placebo randomized in permuted blocks of four. The pharmacy will be responsible for generation of the allocation sequence. Study medicine and placebo are placed in identical opaque boxes and will be numbered randomly according to allocation. Once prepared the pharmacy will ship twenty boxes to the five “physician staffed CCU” stations covering the Capital Region of Denmark. The CCUs are manned 24 h 7 days a week with a physician (henceforth referred to as “CCU physician”) and an experienced paramedic. The responsible project physician will ensure that any CCU station always has boxes in storage and ready for use, and when box storage is used, the pharmacy will provide new boxes. Before each shift, the on-duty physician and paramedic will make sure that a minimum of two boxes are stored in their unit. When a patient is successfully screened the including physician will open one of these boxes and use the content (study medicine or placebo). The box number will be registered by the including physician and for safety reasons a photo of the box number will be taken to the pre-hospital journal and the empty box will be delivered to the department at hospital admission. All treating in-hospital personnel and study coordinators will remain blinded throughout the study. Still, in case of need for emergency unblinding, this can be done by the principal investigator at each site.

#### Concealment mechanism {16b}

Treatment allocation is managed as described above. The opaque boxes containing study medicine or placebo are identical besides the assigned unique allocation ID written on the box. Treatment assignment during the trial is only available for the including CCU physician and the accompanying medical assistant manning the CCU following screening of the patient and contact with the trial guardian for informed consent. Once informed consent is obtained, the including physician opens a random opaque box and infuse the content, i.e., study medicine or placebo. The box allocation number will be available at all time through information filled out in the pre-hospital journal, consent by the including physician and/or the delivery of the used box at the department of admission.

#### Implementation {16c}

Allocation of study medicine and placebo will be done by the pharmacy as described in {16a}. After inclusion and completion of study drug infusion, the including CCU physician will complete an electronic consent through REDCap with allocation number and confirm that inclusion is made following contact and consent from the trial guardian. No in-hospital personnel will have knowledge of allocation at any time and the including physician will not take further part in treatment of the patient. At trial completion the investigators will be informed of allocation.

### Blinding {17}

#### Who will be blinded {17a}

All in-hospital personnel, research personnel, and patients will be blinded to intervention allocation throughout the trial. The including pre-hospital CCU physician and the medical assistant will be blinded during screening, inclusion, and randomization of the patient, but unblinded upon breaking concealment of the randomized box. Infusion is completed before arrival at the hospital and no information other than allocation number will be given at hospital admission. When follow-up is completed, and data collected, the database will be locked and treatment allocation will be unblinded.

#### Procedure for unblinding {17b}

Information on treatment allocation is kept in concealed envelopes in a safe available to authorized personnel at each site. Unblinding will only occur if the knowledge is necessary for treatment of the patient or in case of safety reporting for the involved authorities. In any case of unblinding, the PI will be contacted beforehand. The unblinding will be documented in the trial master file and the patient’s electronic case report fil (eCRF), and the sponsor-investigator will be informed within 1 day.

## Methods: data collection, management, and analysis

### Data collection

#### Assessment of outcomes {18a}

Patient data will be handled as ordinary chart records. All data will be kept according to national legislation, i.e., the General Data Protection Regulation (GDPR) and the Data Protection Act. Acceptance of the data handling and management will be applied for at these institutions before initiation of the study. All handling of personal data in the study is the responsibility of the principal investigator. The study database will be constructed in accordance with national legislation and local practice. Data is entered by study personnel and the database will be maintained for 15 years and anonymized if requested by relevant authorities. Data will be monitored by the Good Clinical Practice (GCP) unit, including review of consent forms and eligibility for the trial. The frequency of onsite monitoring will depend on compliance with the protocol, number of enrolled patients, and the quality of data handling. The principal investigator will be responsible for all data in the eCRF. At completion of the study, biomarkers will be analyzed, and data will be stored in an encrypted server with a backup function.

#### Participant retention and follow-up plan {18b}

Patients included are expected to remain in the ICUs the first three days of admission until the primary endpoints are collected. If a patient awakes and is transferred to the ward, blood sampling will be done here, including samples for the research biobank to analyze the primary endpoints. If a patient is transferred to a local hospital within three days of admission, samples for the biobank are not taken but standard biochemistry will be taken routinely and will be collected. At both sites, dedicated staff will be responsible for follow-up in the ambulatory setting at approximately 90 days following OHCA. The patient will be offered a visit at home if an ambulatory visit is not possible or not wanted by the patient. Patients who discontinue the study during follow-up will remain in the study database if an informed consent is obtained. After 180 days patients will be evaluated through a telephone interview.

### Data management {19}

The database system REDCap® is used for data storage. REDCap is hosted and maintained by the Regional IT department, and data quality will be monitored as described in the “ Assessment of outcomes {18a}” section.

### Confidentiality {27}

Patient confidentiality will be ensured throughout the trial according to national and international legislation (GDPR and the Data Protection Act). Data entered in REDCap are encrypted and will only be assessed by dedicated personnel. After trial completion, the biobank material analyses will be stored in a secured server, and the results from the trial will only be available upon request if approval is provided by relevant authorities.

### Plans for collection, laboratory evaluation, and storage of biological specimens for genetic or molecular analysis in this trial/future use {33}

A research biobank is established from trial initiation and biological material is stored according to legislation and approvals. The material will be analyzed at an advanced laboratory following inclusion of the last participant. Currently, no genetic studies are planned, and future studies including the material are only possible with approval from relevant authorities.

### Statistical methods

#### Statistical methods for primary and secondary outcomes {20a}

Analyses of primary and secondary outcomes will be conducted on the intention-to-treat population. Categorical variables will be presented as numbers and continuous variables will be presented as mean (± SD) or median (25th percentile–75th percentile), according to distribution. The primary analyses of the co-primary endpoint will include the modified intention-to-treat population.

Further, we will conduct sensitivity analyses of the co-primary endpoint including all randomized patients. In this analysis, values missing because a patient died prior to blood sampling will be replaced with the median value of the given biomarker in patients dying after blood sampling, but before 30 days.

Continuous endpoints, including the co-primary endpoint, will be assessed at multiple time points and analyzed by application of linear mixed models of covariance. For approximation of normal distribution, logarithmic transformation will be done, as appropriate. The application of linear mixed models will ensure that a higher power is achieved compared to the sample size calculation, which is based on a single measurement (see the “Sample size {14}” section). For analyses of categorical secondary outcomes, chi-squared or Fisher’s exact test will be applied.

Cox proportional hazard models will be applied and sequentially adjusted for the interaction between treatment arm and relevant variables (sex, age, primary ECG rhythm, time to ROSC, pPCI performed, NSE-, and IL-6 levels). Differences in mortality between treatment arms will be depicted in Kaplan–Meier estimate plots and compared with the log-rank test.

R Studio, version 1.2.5001, will be used for all analyses (RStudio Team [2020]. RStudio: Integrated Development for R. RStudio, PBC, Boston, MA; URL: http://www.rstudio.com/).

#### Interim analyses {21b}

For monitoring of general safety, interim analyses of mortality and hospital length of stay will be performed after 60 participants have been included in the trial.

#### Methods for additional analyses (e.g., subgroup analyses) {20b}

Additional subgroup analyses will be performed on patients with ST-elevation myocardial infarction (primarily suspected in the ECG followed by pPCI) to decide whether active study drug, methylprednisolone, can mitigate the cardiac injury.

Another subgroup will be patients not intubated at admission since a part of included patients will wake up sufficiently, so intubation is not needed and therefore not go to the ICU. Primarily, the changes in markers of inflammation will be compared according to allocation to study drug or placebo and secondarily awake patients will be compared with comatose patients.

#### Methods in analysis to handle protocol non-adherence and any statistical methods to handle missing data {20c}

All analyses will be made on the modified intention-to-treat population. For missingness greater than 10% for the co-primary endpoint, multiple imputations by chained equations will be applied as sensitivity analysis with generation of 10 individual data sets.

#### Plans to give access to the full protocol, participant level-data, and statistical code {31c}

Both protocol and data will be available upon reasonable request and approval from relevant authorities after the trial has been completed.

### Monitoring

#### The coordinating center and trial steering committee {5d}

The trial is anchored at Rigshospitalet as the coordinating center. The trial steering committee (SC) will consist of LO, CH, JK, and FF and will represent both participating centers (Rigshospitalet and Gentofte Hospital). Composition of the data monitoring committee (DMC) is described in {21a}. The local GCP-unit will monitor the trial according to national and international guidelines.

#### The data monitoring committee {21a}

The DMC will consist of Professor Lars Køber, Department of Cardiology, Rigshospitalet and Professor Anders Perner, Department of Anesthesiology, Rigshospitalet. Interim analyses and monitoring of overall conduct of the trial will be performed by the DMC and decisions are reported to the sponsor. The DMC is independent of the sponsor and has no competing interests that could impact the trial.

#### Harms: adverse events {22}

AEs will be recorded daily for the first seven days and further entered in a pre-specified form in the eCRF. AEs occurring after day seven will be evaluated at 30- and 180-day follow-up. For each AE, relationship to trial intervention will be rated as “probable,” “possible,” “unlikely,” or “unknown.” Trial investigators will record and evaluate SAEs and SUSARs throughout the trial. If a life-threatening or fatal SUSAR occurs, the Danish Medicine Agency (DMA) will be notified by the sponsor within seven days. Further, the sponsor will inform the DMA on all follow-up action to these SUSARs no more than 8 days after the reporting. Any other SUSARs will be reported to the DMA no later than 15 days from when the sponsor is informed. For assessment of potential causality between the trial intervention, i.e., whether the adverse event is suspected, the summary of product characteristics for Solumedrol will be used. All SAEs occurring at both trial centers will be submitted once a year by the sponsor and a safety report of all trial patients will be submitted to the DMA.

No later than 90 days after the trial has been completed, the sponsor will notify the DMA. If the trial is stopped earlier than planned, the reasons will be reported.

In addition, we will report all SAEs as endpoint measures and all SUSARs in the final trial report and the results of the trial will be reported on EudraCT.

#### Auditing {23}

The DMC will perform interim analyses after 60 patients have participated in the trial and advice the sponsor and the SC thereafter. The DMC will have unlimited access to data upon request. The GCP monitoring will be externally done by the Copenhagen GCP unit and both on- and off-site monitoring will be performed throughout the trial. Monitoring frequency is decided by the GCP unit according to compliance with the trial protocol. The principal investigators will be responsible for all data entered in the eCRF.

#### Plans for protocol amendments (e.g., trial participants, ethical committees) {25}

Relevant protocol revisions and amendments have been communicated and authorized by all relevant authorities. No changes in the current protocol will be done and updates to the clinical trial registry have been done accordingly (https://clinicaltrials.gov/ct2/show/NCT04624776). The trial status will be updated as appropriate.

#### Dissemination plans {31a}

All trial analyses will be done by the SC, and the trial will be unblinded upon acceptance from the committee. Authorship will be granted according to ICMJE guidelines. The main results of the trial will be published in a peer-reviewed international journal regardless of the results and further presented at international congresses. Trial results will be communicated to participating patients and relevant patient relatives if this was requested in the informed consent form.

## Discussion

The aim of this study is to investigate whether an early infusion of 250 mg methylprednisolone can mitigate the systemic inflammatory response seen in comatose patients resuscitated after OHCA. The co-primary endpoints investigated are the biomarkers IL-6, and NSE and we expect to find a reduction of 20% for both biomarkers measured daily from admission to 72 h following admission.

Inflammation has a key role in the complex systemic response, PCAS. Previous findings have shown that circulating levels of inflammatory biomarkers such as IL-6 are associated to outcome following OHCA, i.e., neurological function and death [9, 31]. A possible clinical benefit of early anti-inflammatory treatment in OHCA remains unknown, but several studies targeting inflammation have promising results. Two small studies from 2009 and 2013 found improved survival and neurological outcome following a combination of epinephrine, vasopressin, and glucocorticoid in patients suffering from IHCA [24, 25]. In both of these studies, glucocorticoid intervention was a low dose of 40 mg methylprednisolone injected during CPR and subsequently patients who developed shock were treated with stress-dose hydrocortisone for a maximum of 7 days. Further, a similar recent Danish study in IHCA patients compared the combination of vasopressin and methylprednisolone (40 mg) with placebo administered during CPR and found the combination to be associated with a higher rate of ROSC, but not survival [26]. The most important difference from these studies compared to the present study was the timing (intra-arrest administration) and dosage (40 mg VS 250 mg) of glucocorticoid. The populations studied consisted of IHCA patients, which in a recent observational study has been found to be similar to OHCA patients in demographics, but with a higher burden of cardiovascular comorbidity [32]. Based on current evidence, ILCOR and the European Resuscitation Council do not support the use of glucocorticoids neither as intra-arrest- or post-cardiac arrest treatment in IHCA and OHCA [33, 34]. However, further evidence could clarify the optimal use and possible role of glucocorticoids in cardiac arrest treatment.

Several studies have demonstrated the molecular mechanisms of glucocorticoids, and it is clear that the effects work both through genomic and non-genomic pathways [23, 35], whereas genomic effects alter protein expression within hours, non-genomic effects are believed to kick in within seconds to minutes. A recent review reported rapid non-genomic anti-inflammatory properties both in transformed and immune cells and concluded that these effects are not well understood but could have significant therapeutic value [36]. Further, both genomic and non-genomic effects of glucocorticoids on the cardiovascular system have been suggested to induce an increase in both blood pressure and contractility [37, 38]. Similarly, evidence indicates that glucocorticoids may effectively treat neuroinflammation, which may be comparable to the condition seen in PCAS. Still, the mechanisms are both poorly understood and investigated [39, 40]. As the intervention in this study is a high dose of methylprednisolone injected shortly after ROSC, we hope to achieve essential knowledge about the beneficial non-genomic effects of glucocorticoids by mitigation of the inflammatory part of PCAS at the earliest stage possible and hereby possible improving hemodynamic parameters and neurological stunning. The results of this study should be interpreted as hypothesis-generating in terms of the need to conduct a larger scale study powered towards clinical endpoints such as survival and neurological outcome.

## Trial status

Protocol: v3.1 of September 23, 2021

Recruiting status: First patient included on October 2020. Estimated recruitment completed in July 2022 and completion of 180 day follow up in January 2023.

## Data Availability

Data required to support the protocol are available upon request and approval from relevant authorities.

## Abbreviations

ABG: Arterial blood gas
AE: Adverse event
ALAT: Alanine aminotransferase
ALP: Alkaline phosphatase
AMI: Acute myocardial infarction
ASAT: Aspartate aminotransferase
CCU: Critical care unit
CKMB: Creatine kinase-MB
CPC: Cerebral performance categories
DMA: Danish medicines agency
DMC: Data monitoring committee
ECG: Electrocardiogram
ECRF: Electronic case report file
G-CSF: Granulocyte-colony stimulating factor
GCP: Good clinical practice
GCS: Glasgow coma scale
GDPR: General data protection regulation
GM-CSF: Granulocyte macrophage-colony stimulating factor
HSCRP: High-sensitive C-reactive protein
ICU: Intensive care unit
IFN: Interferon
IHCA: In-hospital cardiac arrest
IL: Interleukin
ILCOR: International liaison committee on resuscitation
INR: International normalized ratio
MCP-1: Monocyte chemoattractant protein-1
MIP-1: Macrophage inflammatory protein-1
MOCA: Montreal cognitive assessment
MRS: Modified Rankin score
NFL: Neurofilament light chain
NSE: Neuron specific enolase
OHCA: Out-of-hospital cardiac arrest
PCAS: Post-cardiac arrest syndrome
PPCI: Primary percutaneous coronary intervention
ROSC: Return of spontaneous circulation
SAE: Serious adverse event
SC: Steering committee
STEMI: ST elevation myocardial infarction
SUSAR: Suspected unexpected adverse reaction
TEG: Thromboelastographic
TNF: Tumor necrosis factor
TNI: Troponin I
TNT: Troponin T
TOR: Termination of resuscitation
TTE: Transthoracic echocardiography
VBG: Venous blood gas
